# Epicardial Fat Thickness and Bone Mineral Content: The Healthy Twin Study in Korea

**DOI:** 10.2188/jea.JE20170027

**Published:** 2018-05-05

**Authors:** Dong-won Lee, Min Soo Cho, Eun Yeong Choe, Seung Woo Park, Yun-Mi Song, Sang-Chol Lee, Joohon Sung

**Affiliations:** 1 Department of Internal Medicine, Division of Gastroenterology, Korea University Ansan Hospital, Ansan, Korea; 2 Heart Institute, Asan Medical Center, University of Ulsan College of Medicine, Seoul, Korea; 3 Department of Internal Medicine, Division of Endocrinology, Yonsei University College of Medicine, Seoul, Korea; 4 Department of Cardiology and Cardiovascular Imaging Center, Cardiac and Vascular Center, Samsung Medical Center, Sungkyunkwan University School of Medicine, Seoul, Korea; 5 Department of Family Medicine, Samsung Medical Center, Sungkyunkwan University School of Medicine, Seoul, Korea; 6 Department of Epidemiology, School of Public Health, Seoul National University, Seoul, Korea; 7 Institute of Health and Environment, Seoul National University, Seoul, Korea; 8 Bio-MAX Institute, Seoul National University, Seoul, Korea

**Keywords:** epicardial fat, visceral fat, bone mineral content

## Abstract

**Background:**

The conventional concept of positive association between general obesity and bone health was challenged in recent studies reporting the different effects of specific fat deposition on bone health. In the present study, we investigated the association between epicardial fat and bone health.

**Methods:**

We measured echocardiographic epicardial fat thickness (EFT) and bone mineral content (BMC) in a twin-family cohort of Koreans (*n* = 1,198; 525 men, 460 pre- and 213 post-menopausal women). A total 121 pairs of monozygotic twin (MZ) and 404 pairs of dizygotic twin and sibling pairs (DZ/Sib) were included.

**Results:**

EFT was positively associated with BMC in total, as well as in three subgroups (β = 0.107, 0.076, and 0.058 for men, pre-, and post-menopausal women, respectively). The positive association between EFT and BMC remained for DZ/Sib difference analysis, but was absent for MZ comparisons. The positive association between BMI and BMC was consistent for DZ/Sib and MZ difference analysis. After adjusting for the effect of general obesity via BMI, the association between BMC and EFT was statistically non-significant (β = 0.020, 0.000, and −0.009 for men, pre-, and post-menopausal women, respectively).

**Conclusion:**

Our findings do not support epicardial fat’s beneficial effects on bone health, whereas general adiposity has an osteotropic effect. The association between EFT and BMC is through common genetic component factors.

## INTRODUCTION

Obesity and osteoporosis are significant public health problems with increasing prevalence and substantial economic burdens in most industrialized countries.^1^^–^^4^ Although many studies have reported a relationship between bone health and obesity, the association is still inconclusive. Previous epidemiologic studies have demonstrated that higher body mass index (BMI) and body weight had protective roles against bone loss, and weight reduction was associated with significant bone loss.^5^^–^^7^ In this setting, obesity and fat seems to have beneficial effect on bone health in contrast to the detrimental effect on most health conditions, especially on cardiovascular and metabolic diseases.^8^^,^^9^ However, since anthropometric data, such as BMI and body weight, reflect lean mass as well as fat mass (FM), the actual effect of the fat tissue on bone heath is not clear. Therefore, the role of fat on bone health needs to be investigated with a new indicator.

Several recent studies have been shedding new light on the complex bone-fat connection. Adipocytes and osteoblasts seem to interact and reciprocally modulate at many levels, including the human mesenchymal stem cells in the bone marrow, from which they both originate.^10^^,^^11^ In addition, a recent publication demonstrated that different regional fat depots have different impacts on bone health. For example, visceral adipose tissue (VAT) might have detrimental effect on bone mass, in contrast to the beneficial effect of subcutaneous adipose tissue (SAT).^12^^,^^13^ Therefore, the association of bone health with more specific VAT might help to elucidate the observed osteogenic nature of obesity.

Epicardial adipose tissue (EAT) is located between the myocardium and the visceral pericardium, and has the same embryologic origin as intraabdominal mesenteric and omental fat cells.^14^ Its measurement using transthoracic echocardiography (TTE) is simple and is known to be a strong predictor of abdominal VAT.^15^^,^^16^ However, compared with other VAT, EAT contain more smaller adipocytes and secrete more bioactive metabolites, such as adipokines.^17^^,^^18^ Recent study reported an inverse relationship between EAT and bone mineral density (BMD) in acromegaly patients, no studies have evaluated this association in the general population.^19^

In the present study, we aimed to investigate the association between bone health and EAT, as well as other well-known VAT markers, including anthropometric data and regional FM measured using dual-energy X-ray absorptiometry (DXA).

## MATERIALS AND METHODS

### Study design and population

The subjects included in this analysis were participants in the Healthy Twin Study, which was a nationwide population-based cohort study implemented as a part of the Korean genome epidemiology study. It was initiated in 2005, and participants continue to receive follow-up examinations every 3 years. Participants consisted of a twin pair and their first-degree family members. All participants received medical examinations and completed detailed questionnaires about lifestyle and epidemiologic information at one of three medical school-affiliated hospitals. Details on the study design and protocol were previously published.^20^

Among the initial 1,467 subjects who completed an echocardiogram and body composition measurements between 2006 and 2008, 269 subjects were excluded: 220 for poor echocardiographic image quality, such as poor echo window or angle difference, and 49 for a treatment history of osteoporosis. A total of 1,198 subjects (525 men, 460 premenopausal women, and 213 postmenopausal women) were included in our final analysis with monozygotic twin pairs (MZ) (*n* = 121 pairs) and pooled dizygotic twin and sibling pairs (DZ/Sib) (*n* = 404 pairs). Women were considered postmenopausal if they had no history of menstruation during the previous year and fulfilled at least one of the following criteria: natural menopause, use of estrogen replacement therapy, or age older than 50 years.^21^^,^^22^ Natural menopausal women was defined as those with at least 12 consecutive months of amenorrhea not due to surgery and other obvious cause, such as medical treatment or breastfeeding, which is mainly caused by loss of ovarian function. In fact, surgical menopausal women may have normal ovarian function. Therefore, in present study, women with surgical amenorrhea were considered to be postmenopausal women only if they fulfilled the history of bilateral oophorectomy or estrogen replacement therapy or they were 50 years of age or older, which can explain the loss of ovarian function.

All participants provided written informed consent. The study protocol was approved by the Institutional Review Board at Seoul National University School of Public Health.

### Measurement of epicardial fat thickness

Subjects underwent TTE according to standard techniques in the left lateral decubitus position, using commercially available instruments (Vivid E9; GE Healthcare, Horten, Norway). The images were recorded in a digital database. The measurement of epicardial fat thickness was performed by one internist and one cardiologist using an offline DICOM (Digital imaging and Communications in Medicine) viewer (Onis 2.5 professional version; Digital Core, Tokyo, Japan). They were unaware of the subjects’ clinical information.

**Figure 1.**
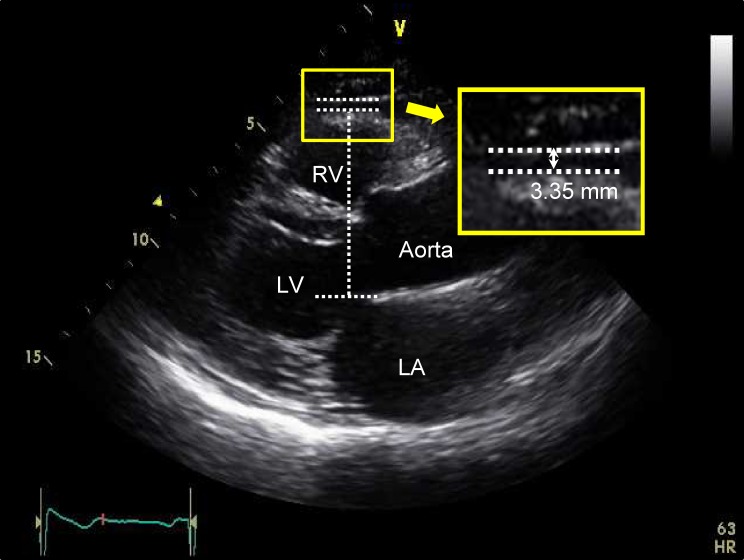
Method of measuring epicardial fat thickness. Epicardial fat thickness was measured perpendicularly on the free wall of the right ventricle from parasternal long axis view at end-systole. LA, left atrium; LV, left ventricle; RV, right ventricle.

Epicardial fat was identified as the echo-free space between the myocardium and the visceral epicardium, and its thickness was measured perpendicularly on the free wall of the right ventricle at end-systole from the standard parasternal long axis view.^23^^,^^24^ In order to standardize the measurements between the observers, the aortic annulus was used as an anatomical landmark and the epicardial fat thickness was measured at the point on the free wall of the right ventricle along the midline of the ultrasound beam, perpendicular to the aortic annulus (Figure 1). The intra- and inter-observer correlations for the measurement of epicardial fat thickness were good, with intraclass correlation coefficients of 0.95 (range, 0.93–0.97) and 0.92 (range, 0.88–0.95), respectively (eFigure 1).

### Measurement of anthropometric data and body composition

Body weight and height were measured according to standard methods while the subjects were wearing a light gown or light indoor clothing. Minimum waist circumference (WC) was measured in the standing position at the point between the lower rib margin and the iliac crest. Hip circumference was measured as the largest circumference over the buttock. BMI (kg/m^2^) was calculated as weight divided by height squared, and the waist-to-hip ratio (WHR) was calculated as WC divided by hip circumference. Whole body bone mineral content (BMC; kg), BMD (g/cm^2^) of the whole body, the lumbar spine, and the pelvis; whole-body mass, regional FM (kg), and lean mass (LM; kg) were measured using DXA (Delphi W; Hologic, Boston, MA, USA).

### Clinical information

The following clinical and demographic data were extracted from each patient’s baseline questionnaire: past medical history of chronic diseases, including hypertension, diabetes mellitus, hyperthyroid disease, and osteoporosis; female reproductive history, including age at menopause and use of estrogen replacement therapy; and information about cigarette smoking, alcohol consumption, and exercise habits.

### Statistical analysis

The value of each continuous variable was expressed as a mean (standard deviation [SD]). Each categorical or discrete variable was presented as a percentage. Comparisons among the groups (men, premenopausal women, and postmenopausal women) were performed using analysis of variance, analysis of covariance (ANCOVA), and the Chi-squared (χ^2^) test. Multiple comparisons between two groups were performed using post hoc analysis. The relationships between the epicardial fat thickness (EFT) and other measures of body composition were analyzed using Pearson’s or Spearman’s correlation analysis. Linear mixed models in each group were used to evaluate associations between EAT and bone mass, and to correct for familiar interdependence. Age and height were included as covariates in regression model 1, and past medical history (hypertension, diabetes mellitus, and thyroid disease) as well as behavioral factors (smoking, alcohol, and exercise habits) were added as covariates in model 2. In order to evaluate the exact relationship between EAT and bone mass, general obesity markers, such as BMI (model 3) or total FM (model 4), were added as covariates for excluding general obesity effect of fat on bone. Age, height, BMI, past medical history variables, and lifestyle variables were adjusted as fixed effects, and each family and twin unit was adjusted as a random effect in our linear mixed models.

Additionally, we conducted within-pair analysis for MZs, same-sex DZs, and age adjusted same-sex sibling pairs. By comparing general population, MZ and DZ/sibling, we could gain further insight into the nature of the association, particularly that of genetic correlation or environmental correlation.^25^^,^^26^ Because MZ shares 100% of genetic information, any meaningful differences within MZ pairs are reasonably interpreted as non-genetic contributions including epigenetics. On the other hand, if associations in general populations are materially weakened or nullified in the MZ comparisons, it strongly suggests that the associations might stem from common genetic grounds. These findings are further supported by the positive associations in the DZ pair comparisons, who share only 50% of genetic constitutions but similar level of environmental sharing. In the present study, differences in obesity indices within pairs were compared with the differences in the BMC within the same pairs using Spearman’s correlation coefficient.

Statistical analyses were performed using Statistical Package for the Social Sciences statistical software (version 18; SPSS-IBM, Chicago, IL, USA) or by R version 3.02 (R Foundation for Statistical Computing, Vienna, Austria). All tests were two-tailed, and a *P*-value <0.05 was considered statistically significant.

## RESULTS

The baseline characteristics of the subjects are listed in Table 1. The body composition parameters and clinical information were statistically different according to sex and menopausal status. Men had higher BMI, WC, WHR, and LM than women. Fat-related parameters, such as total FM and trunk FM, were lower in men than in women, with the exception of head FM. Postmenopausal women had more total fat and trunk fat, but had lower leg fat and LM than premenopausal women. EFT was highest in postmenopausal women and lowest in premenopausal women. BMC was higher in men—because of their larger body size—than in women. However, the difference in BMD between the sexes was small, and no significant difference in spine BMD was identified between men and premenopausal women. Hypertension and diabetes mellitus were most prevalent in postmenopausal women; risky heath behaviors, such as smoking and drinking alcohol, were the highest in men.

**Table 1.** Baseline characteristics of the study population

Variables	Male (*n* = 525)	Premenopausal (*n* = 460)	Postmenopausal (*n* = 213)
Age, years	44.2 (14.7)	35.5 (8.4)	56.2 (8.1)
Epicardial fat thickness, mm	1.93 (0.72)	1.73 (0.72)	2.17 (0.81)
BMC, whole body, kg	2.51 (0.40)	2.04 (0.33)	1.80 (0.31)
BMD, whole, g/cm^2^	1.17 (0.13)	1.11 (0.19)	1.03 (0.12)
BMD, spine, g/cm^2^	0.98 (0.17)	0.98 (0.13)	0.88 (0.19)
BMD, pelvis, g/cm^2^	1.15 (0.16)	1.11 (0.13)	1.06 (0.20)
Height, cm	170.2 (8.5)	158.2 (9.5)	155.2 (5.4)
Weight, kg	71.6 (10.5)	57.2 (9.2)	58.4 (8.5)
BMI, kg/m^2^	24.5 (2.9)	22.6 (3.2)	24.2 (3.1)
Waist circumference, cm	85.7 (7.9)	76.1 (8.2)	81.7 (8.4)
Waist-to-hip ratio	0.91 (0.21)	0.84 (0.06)	0.90 (0.06)
Fat mass, kg	16.0 (5.4)	17.9 (5.4)	19.8 (5.1)
Fat mass, %	22.5 (5.4)	31.4 (6.1)	34.4 (5.0)
Trunk fat mass, kg	8.7 (3.3)	8.5 (3.3)	10.5 (3.2)
Head fat mass, kg	1.2 (0.2)	1.0 (1.1)	1.0 (0.1)
Leg fat mass, kg	4.4 (1.6)	6.3 (1.7)	5.8 (1.6)
Soft lean mass, kg	52.4 (6.6)	37.2 (4.7)	36.0 (3.9)
Hypertension, %	17.0	2.6	27.7
Diabetes mellitus, %	6.9	1.1	8.5
Hyperthyroidism, %	0.6	2.6	0.9
Smokers, %	67.4	12.8	5.2
Drinkers, %	85.0	74.8	45.1
Regular exercise, %	41.7	30	40.8

BMC, bone mineral content; BMD, bone mineral density; BMI, body mass index.

^a^Data are expressed as means (SD).

Table 2 and eFigure 2 show the correlations of EFT with anthropometric and body composition variables. EFT was highly associated with body FM, especially trunk FM, in postmenopausal women. EFT was also associated with classical abdominal visceral obesity parameters, such as WC and WHR. EFT was positively correlated with age, while BMC was negatively correlated with age. BMC showed a strong positive correlation to height.

**Table 2.** Associations between epicardial fat thickness and body composition variables

	Male		Premenopausal		Postmenopausal		Total	
	*r*	*P*-value	*r*	*P*-value	*r*	*P*-value	*r*	*P*-value
Total fat mass	0.368	0.000	0.392	0.000	0.484	0.000	0.392	0.000
Trunk fat mass	0.375	0.000	0.388	0.000	0.495	0.000	0.423	0.000
Arms fat mass	0.341	0.000	0.336	0.000	0.407	0.000	0.340	0.000
Legs fat mass	0.262	0.000	0.301	0.000	0.340	0.000	0.220	0.000
Head fat mass	0.241	0.000	−0.037	0.451	0.274	0.000	0.009	0.765
Height	0.008	0.854	0.069	0.141	−0.060	0.387	0.023	0.422
Weight	0.361	0.000	0.393	0.000	0.470	0.000	0.338	0.000
Waist	0.469	0.000	0.393	0.000	0.496	0.000	0.444	0.000
Hip	0.298	0.000	0.317	0.000	0.444	0.000	0.326	0.000
BMI	0.473	0.000	0.437	0.000	0.553	0.000	0.519	0.000
Waist-to-hip ratio	0.058	0.306	0.390	0.000	0.611	0.000	0.163	0.000
Soft lean mass	0.136	0.016	0.401	0.000	0.418	0.000	0.204	0.000

BMI, body mass index.

^a^Data presented are Pearson’s correlation coefficients (*r*).

We also examined the differences in whole-body BMC, whole-body BMD, and body-part specific BMD across the tertiles of EFT, adjusting for age and height in all three subgroups using the ANCOVA test. As shown in Table 3, BMC significantly increased across increasing tertiles of EFT, especially in men and postmenopausal women. This tendency was not observed in whole-body or spine BMD, particularly in women. The results of the post hoc analysis between tertiles are shown in eTable 1.

**Table 3.** Comparisons of the least squares means of bone mineral content and bone mineral density according to epicardial fat thickness tertiles adjusted for age and height

	1^st^ tertile	2^nd^ tertile	3^rd^ tertile	*P-value* for trend
**Men**				
BMC, whole, kg	2.41 (0.03)	2.51 (0.03)	2.59 (0.03)	0.000
BMD, whole, g/cm^2^	1.15 (0.01)	1.18 (0.01)	1.19 (0.01)	0.037
BMD, spine, g/cm^2^	0.98 (0.01)	0.99 (0.01)	0.98 (0.01)	0.862
BMD, pelvis, g/cm^2^	1.12 (0.01)	1.15 (0.01)	1.18 (0.01)	0.024
**Premenopausal women**				
BMC, whole, kg	1.99 (0.03)	1.99 (0.03)	2.08 (0.03)	0.058
BMD, whole, g/cm^2^	1.11 (0.01)	1.10 (0.01)	1.13 (0.01)	0.317
BMD, spine, g/cm^2^	0.97 (0.01)	0.98 (0.01)	0.98 (0.01)	0.665
BMD, pelvis, g/cm^2^	1.08 (0.01)	1.10 (0.01)	1.13 (0.01)	0.002
**Postmenopausal women**				
BMC, whole, kg	1.73 (0.03)	1.80 (0.03)	1.89 (0.03)	0.003
BMD, whole, g/cm^2^	1.02 (0.01)	1.04 (0.02)	1.06 (0.01)	0.104
BMD, spine, g/cm^2^	0.86 (0.02)	0.89 (0.02)	0.89 (0.02)	0.539
BMD, pelvis, g/cm^2^	1.04 (0.02)	1.05 (0.02)	1.08 (0.03)	0.568
**Total**				
BMC, whole, kg	2.14 (0.02)	2.18 (0.02)	2.27 (0.02)	0.000
BMD, whole, g/cm^2^	1.11 (0.01)	1.12 (0.01)	1.15 (0.01)	0.006
BMD, spine, g/cm^2^	0.96 (0.01)	0.95 (0.01)	0.97 (0.01)	0.430
BMD, pelvis, g/cm^2^	1.09 (0.01)	1.11 (0.01)	1.15 (0.01)	0.000

BMC, bone mineral content; BMD, bone mineral density.

^a^Data are expressed as means (SE).

**Figure 2.**
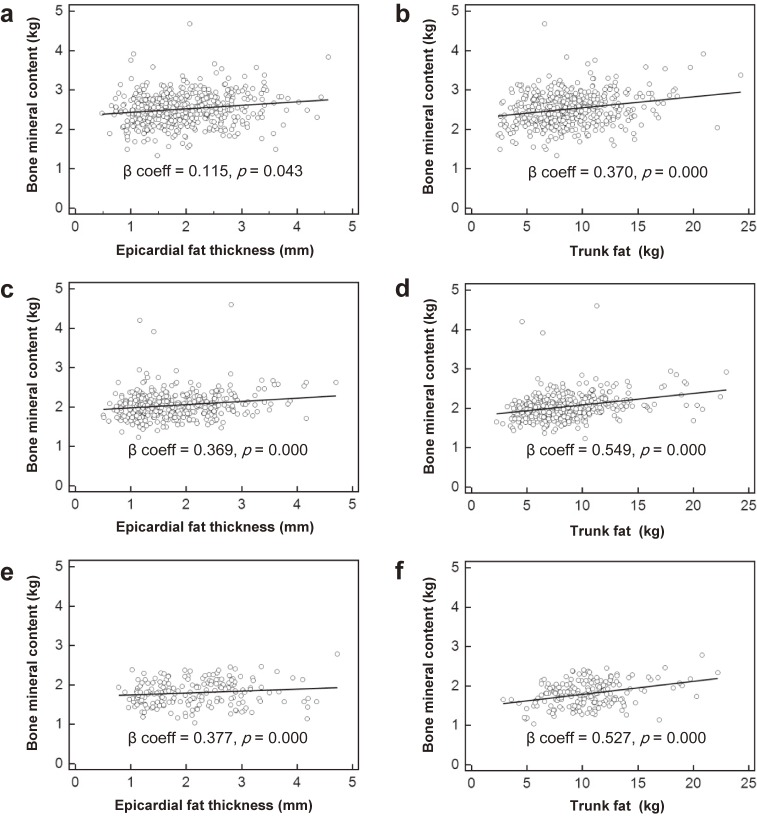
Association between BMC and epicardial fat thickness or trunk fat a, b: Men; c, d: premenopausal women; e, f: postmenopausal women. BMC, bone mineral content.

Figure 2 shows the association between BMC and EFT and trunk fat. In bivariate unadjusted analyses, BMC increased with increasing EFT in all subgroups. These tendencies were also observed in associations between BMC and trunk fat in all subgroups.

Table 4 shows the multivariate-adjusted associations between BMC and body composition variables after correcting for familial interdependence. EFT was positively associated with BMC in men, premenopausal women, and postmenopausal women in the age- and height-adjusted model (model 1). This association was consistent after controlling for past medical history (hypertension, diabetes, and hyperthyroid disease) and behavioral factors (smoking, alcohol, and exercise habits; model 2). Trunk FM and classical indices of abdominal obesity parameters, including WHR, were also positively associated with BMC in both models. The same associations were observed with total FM. Model 3 shows the associations between BMC and EFT and other body composition variables after adjusting for general obesity with BMI. The association between BMC and EFT was statistically insignificant in all groups. In model 4, which adjusted for general obesity using total FM, there was a significant positive correlation between epicardial fat and BMC in men, but no statistical significant association was observed in premenopausal women or postmenopausal women. However, the association between BMC and the conventional abdominal obesity markers, including WHR and trunk FM converted to negative after additionally adjusting for BMI.

**Table 4.** Multivariate-adjusted associations between bone mineral content and body composition variables using a linear mixed model

	Men		Premenopausal		Postmenopausal	
	β (SE)	*P*-value	β (SE)	*P*-value	β (SE)	*P*-value
**Model 1**						
EFT	111 (22)	0.000	74 (23)	0.001	89 (22)	0.000
WHR	54 (15)	0.000	106 (41)	0.009	189 (52)	0.000
Trunk fat mass	27 (5)	0.000	25 (5)	0.000	32 (6)	0.000
Total fat mass	19 (3)	0.000	17 (3)	0.000	20 (4)	0.000
Soft lean mass	39 (3)	0.000	48 (4)	0.000	46 (7)	0.000
**Model 2**						
EFT	107 (21)	0.000	76 (22)	0.001	58 (20)	0.004
WHR	376 (51)	0.000	177 (47)	0.000	220 (51)	0.000
Trunk fat mass	30 (5)	0.000	27 (5)	0.000	30 (5)	0.000
Total fat mass	20 (3)	0.000	17 (3)	0.000	20 (3)	0.000
Soft lean mass	45 (4)	0.000	51 (5)	0.000	49 (8)	0.000
**Model 3**						
EFT	20 (24)	0.408	0 (23)	0.996	−11 (25)	0.666
WHR	−256 (95)	0.008	−96 (44)	0.031	−161 (87)	0.067
Trunk fat mass	−31 (7)	0.000	−29 (8)	0.000	−15 (10)	0.150
Soft lean mass	35 (6)	0.000	44 (9)	0.000	22 (12)	0.060
**Model 4**						
EFT	80 (24)	0.001	31 (22)	0.149	19 (25)	0.445
WHR	1,550 (939)	0.101	−553 (354)	0.120	−787 (967)	0.417
Trunk fat mass	−22 (22)	0.305	0 (18)	0.990	−7 (18)	0.714
Soft lean mass	39 (4)	0.000	40 (5)	0.000	33 (10)	0.001

BMC, bone mineral content; EFT, epicardial fat thickness; WHR, waist-to-hip ratio; SE, standard error.

^a^Model 1: The fixed effects (age and height) and the random effect (each family and twin unit) were adjusted.

^b^Model 2: Model 1 + additional adjustments as fixed effects for hypertension, diabetes, hyperthyroid disease, smoking habits, alcohol consumption, and regular exercise.

^c^Model 3: Model 1 + BMI.

^d^Model 4: Model 1 + total fat mass.

^e^The unit of beta coefficient for BMC and epicardial fat thickness is gram/mm; Waist-to-hip ratio and BMC, gram/0.1 unit; trunk fat mass and BMC, gram/kilogram; soft lean mass, gram/kilogram.

Table 5 shows the within-pair analysis in MZ and DZ/Sib. The positive association between EFT and BMC was consistent in DZ/Sib difference analysis, while within-pair difference analysis for MZ, which shared 100% of their genetic constitutions, cancelled this association. This strongly suggested an association of genetic nature between the BMC and EFT. Contrary to EFT, the positive association between BMI and BMC was consistent for both DZ/Sib and MZ in difference analysis, indicating either genetic or environmental associations. The results according to sex and menopausal status in within-pair analysis are shown in eTable 2.

**Table 5.** The correlation between epicardial fat thickness or body mass index difference, and bone mineral content difference in MZ and DZ/Sib

	DZ/Sib difference analysis (*n* = 404 pairs)		MZ difference analysis (*n* = 121 pairs)	
	
	BMC difference	*P*-value	BMC difference	*P*-value
Epicardial fat difference	0.205	0.000	0.088	0.339
BMI difference	0.425	0.000	0.367	0.000

BMC, bone mineral content; BMI, body mass index; DZ, dizygotic twins; MZ, monozygotic twins.

^a^Data presented are Spearman’s correlation coefficients.

^b^DZ/Sib difference: pooled same-sex dizygotic twins and age-adjusted same-sex sibling pairs, where the pairwise differences in obesity measures were regressed on the differences in the BMC of the same pairs.

^c^MZ difference: same analysis was conducted for monozygotic twin pairs.

Approximately 15% of the initial subjects were excluded from analysis due to poor echocardiographic images that affected measurement reliability (eFigure 3). These subjects were younger and had lower total fat (including epicardial fat thickness) and abdominal fat than the subjects with good echocardiographic images (eTable 3). The associations between BMC and EFT did not change when subjects with poor echocardiographic images were included in the analysis. The β coefficient of the association between EFT and BMC in Model 2 was 0.117 for men (*P* = 0.000), 0.076 for premenopausal women (*P* = 0.000), and 0.065 for postmenopausal women (*P* = 0.003).

## DISCUSSION

The results from our study of the Korean Healthy Twin cohort did show that EFT was positively associated with BMC, regardless of sex and menopausal status. However, after additionally adjusting for general obesity, the association was statistically insignificant for most outcomes. Additionally, although abdominal obesity parameters, such as WHR and trunk FM, were positively associated with BMC, this association shifted to a negative or insignificant correlation after adjusting for general obesity with BMI or total FM. In addition, we demonstrated that the association between the BMC and EFT had a significant genetic basis.

The exact association between fat and bone density is still controversial. Several previous studies reported positive associations between body fat and BMC or BMD, and two plausible mechanisms were suggested on the basis of two main characteristics of fat.^27^ One mechanism is related to increased weight bearing of bones, which directly activates adaptive bone remodeling^28^^–^^30^; the other mechanism is associated with paracrine and hormonal effects of fat, which enhance anabolic effects on bone through increased production of sex hormones and hormonal factors, such as insulin, leptin, and amylin.^31^^–^^35^ However, other previous reports demonstrated a negative relationship between fat and BMC or BMD.^7^^,^^36^^–^^38^ In most of these studies, body weight was used as an important covariate in the analysis; however, this may have created a false association between FM and bone mass due to biases from strong co-linearity between FM and body weight.^39^ Therefore, we used height as an important covariate instead of weight; because whole-body BMC and BMD are highly associated with whole-body bone size and height is known to be a good surrogate marker for body size.^27^^,^^36^ Also, the negative association between fat and bone might be partially due to the specific role of different region’s fat on bone. For example, several studies showed that abdominal visceral fat was negatively associated with bone mass, in contrast to the positive association of subcutaneous fat with bone.^12^^,^^13^

Epicardial fat, located only between the myocardium and the visceral pericardium, is known to have considerable systemic effects by secreting bioactive adipokines and being involved in the lipid metabolism, such as in the production of free fatty acid.^40^ Until now, studies on epicardial fat have been focused on cardiovascular diseases and metabolic syndrome, but there have been few studies on the relation between epicardial fat and bone health, although adipokines secreted from the fat are significantly associated with bone metabolism.^41^^,^^42^ A recent study showed an inverse relationship between epicardial fat and BMD of lumbar spine in acromegaly patients, which was mediated by Dickkopf-related protein 1 (DKK1), an inhibitor of osteoblast differentiation and bone formation produced by preadiopocytes and osteocytes in humans.^19^ However, no studies have evaluated this association in the general population.

In the present study, EFT was positively associated with BMC. The association of other conventional abdominal fat variables, including WHR and trunk FM, with BMC was consistent with this result. However, after adjusting for general obesity with BMI, the positive association between EFT and BMC converted to negative, though it was statistically insignificant. In addition, the associations between BMC and conventional abdominal obesity variables also converted to negative. Kim et al reported that abdominal visceral fat had an important role in negatively regulating bone mass if the weight-bearing effect of fat was excluded, which is consistent with our results.^43^ However, given that the directionality between bone, general obesity (or FM), and EFT are still unclear, we cannot exclude the possibility that BMI works as a collider rather than a pure confounder, which our model assumed. But, it is unlikely that bone health status significantly affects BMI or other obesity indices, because fat is metabolically and biologically much more active than the bones and its influences are exerted on virtually all body systems. Bones are also a metabolically active tissue, but the regulations and influences are overwhelmingly toward bone and bone mineral homeostasis. Given this biology, we believe the adjustment of BMI might be insufficient or too simple for testing independent EFT effects, but it is unlikely that BMI works as a collider between EFT and BMC. In addition, considering that the results are similar when adjusting for general obesity using total FM, which does not act as a collider, BMI is also unlikely to act as a collider in this study.

To date, no study addressing the genetic correlation between EFT and BMC has been conducted. Analyzing twins and sibling pairs in the Korean Healthy Twin study cohort, the positive correlation between EFT and BMC was absent in within-pair difference analysis for MZ, which suggests the involvement of genetic constitutions in these findings. It is imperative to conduct further studies investigating the common genetic determinants. In addition, this association suggests that genetically identical individual’s bone health might not benefit from the increase in EFT level.

This study has several strengths. Principally, we used multiple methods for estimating different types of adiposity and covariates that influence BMC. In addition, having a family-and-twin structure enabled us to dissect the associations between fat measures and BMC into those of genetic and non-genetic nature.

However, this study has several limitations. This study had a cross-sectional design, and all participants were Korean. Additionally, echocardiographic EFT may not reflect the exact quantity of total epicardial fat because it is a linear measurement and varies at different locations around the myocardium.

In conclusion, this study suggests that general obesity contributes to the observed positive association between EFT and BMC. The lack of correlation between BMC and EFT independent of BMI or total FM does not support the possible beneficial role of EFT in bone health. However, given the complex inter-correlation between general obesity EFT and bone health, findings from simple adjustment might not preclude an association between EFT and BMC. Moreover, our findings suggest that the observed association between EFT and BMC might involve a genetic correlation.

## APPENDIX A. SUPPLEMENTARY DATA

eFigure 1. The Bland-Altman plots for intra- and inter-observer correlations of epicardial fat thickness.

eFigure 2. Correlogram of epicardial fat thickness with anthopometric and body composition variables. BMC, bone mineral content; BMI, body mass index; Epifat, epicardial fat thickness; WC, waist circumference; WHR, waist to hip ratio.

eFigure 3. The Bland-Altman plots of intra-and inter-observer correlations for epicardial fat thickness in subjects excluded because of poor echocardiographic image quality.

eTable 1. Comparisons of the least squares means of bone mineral content and bone mineral density according to epicardial fat thickness tertiles adjusted for age and height

eTable 2. The correlation between epicardial fat thickness or body mass index difference and bone mineral content difference in MZ and DZ/Sib

eTable 3. Baseline characteristics of the study population according to echocardiographic image quality
