# Supplementary material for: Epicardial Fat Thickness and Bone Mineral Content: The Healthy Twin Study in Korea
Source: J Epidemiol. 2018 May 5;28(5):253–9. doi: 10.2188/jea.JE20170027 (PMC5911676; doi:10.2188/jea.JE20170027)
Supplement: Supplementary file 1 [file je-28-253-s001.pdf]

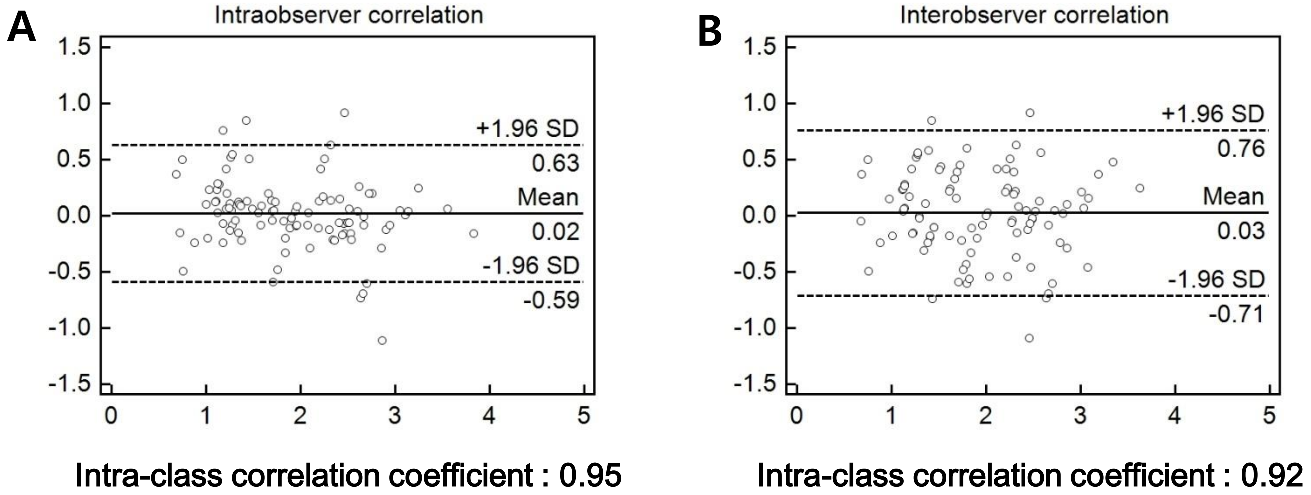

eFigure 1. The Bland-Altman plots for intra- and inter-observer correlations of epicardial fat thickness

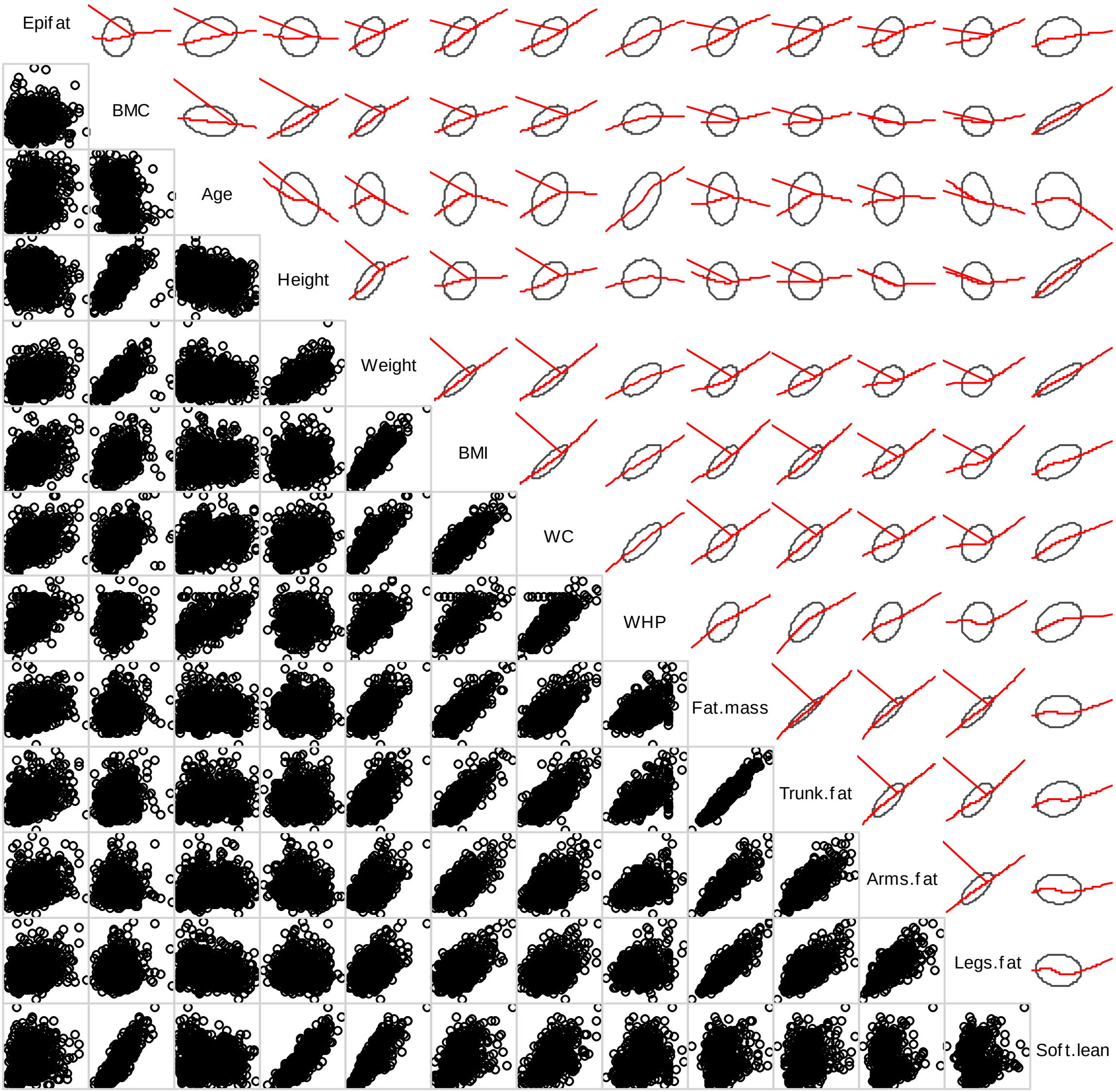

eFigure 2. Correlogram of epicardial fat thickness with anthopometric and body composition variables. BMC, bone mineral content; BMI, body mass index; Epifat, epicardial fat thickness; WC, waist circumference; WHR, waist to hip ratio.

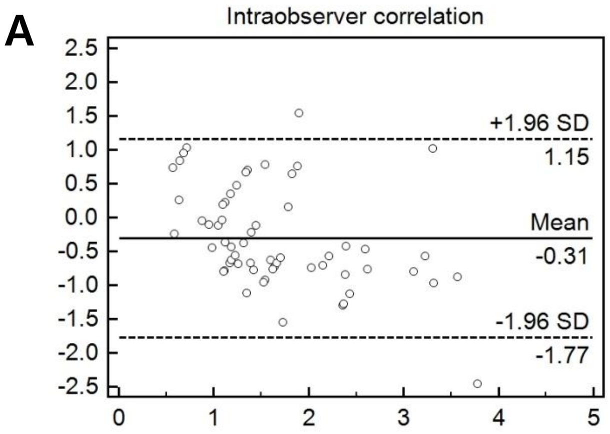

Intra-class correlation coefficient : 0.77

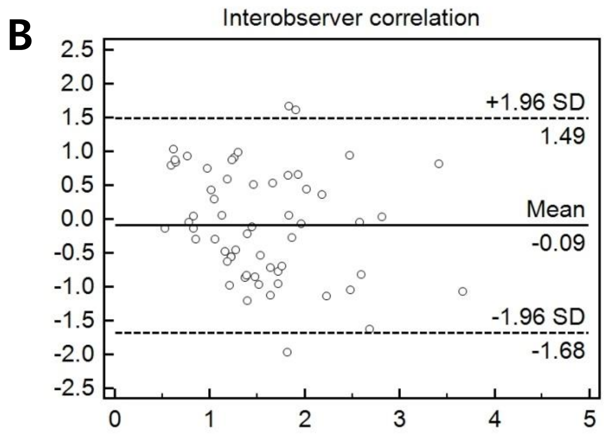

Intra-class correlation coefficient : 0.63

eFigure 3. The Bland-Altman plots of intra- and inter-observer correlations for epicardial fat thickness in subjects excluded because of poor echocardiographic image quality

eTable 1. Comparisons of the least squares means of bone mineral content and bone mineral density according to epicardial fat thickness tertiles adjusted for age and height

	Epicardial fat thickness				
	1st tertile	2nd tertile	3rd tertile	*P-value* for trend	*p*-value
**Male**					
BMC, whole, kg	2.41 (0.03)	2.51 (0.03)	2.59 (0.03)	0.000	1st vs 2nd : 0.008<br>1st vs 3rd : 0.000<br>2nd vs 3rd : 0.074
BMD, whole, g/cm^2	1.15 (0.01)	1.18 (0.01)	1.19 (0.01)	0.037	1st vs 2nd : 0.114<br>1st vs 3rd : 0.011<br>2nd vs 3rd : 0.334
BMD, spine, g/cm^2	0.98 (0.01)	0.99 (0.01)	0.98 (0.01)	0.862	1st vs 2nd : 0.604<br>1st vs 3rd : 0.920<br>2nd vs 3rd : 0.685
BMD, pelvis, g/cm^2	1.12 (0.01)	1.15 (0.01)	1.18 (0.01)	0.024	1st vs 2nd : 0.073<br>1st vs 3rd : 0.000<br>2nd vs 3rd : 0.059
**Premenopausal women**					
BMC, whole, kg	1.99 (0.03)	1.99 (0.03)	2.08 (0.03)	0.058	1st vs 2nd : 0.970

					1st vs 3rd : 0.038
					2nd vs 3rd : 0.038
					1st vs 2nd : 0.577
BMD, whole, g/cm^2	1.11 (0.01)	1.10 (0.01)	1.13 (0.01)	0.317	1st vs 3rd : 0.351
					2nd vs 3rd : 0.133
					1st vs 2nd : 0.750
BMD, spine, g/cm^2	0.97 (0.01)	0.98 (0.01)	0.98 (0.01)	0.665	1st vs 3rd : 0.373
					2nd vs 3rd : 0.560
					1st vs 2nd : 0.254
BMD, pelvis, g/cm^2	1.08 (0.01)	1.10 (0.01)	1.13 (0.01)	0.002	1st vs 3rd : 0.000
					2nd vs 3rd : 0.014
**Postmenopausal women**					
					1st vs 2nd : 0.118
BMC, whole, kg	1.73 (0.03)	1.80 (0.03)	1.89 (0.03)	0.003	1st vs 3rd : 0.001
					2nd vs 3rd : 0.058
					1st vs 2nd : 0.249
BMD, whole, g/cm^2	1.02 (0.01)	1.04 (0.02)	1.06 (0.01)	0.104	1st vs 3rd : 0.034
					2nd vs 3rd : 0.316
BMD, spine, g/cm^2	0.86 (0.02)	0.89 (0.02)	0.89 (0.02)	0.539	1st vs 2nd : 0.370

					1st vs 3rd : 0.310
					2nd vs 3rd : 0.891
					1st vs 2nd : 0.908
BMD, pelvis, g/cm^2	1.04 (0.02)	1.05 (0.02)	1.08 (0.03)	0.568	1st vs 3rd : 0.330
					2nd vs 3rd : 0.387
**Total**					
					1st vs 2nd : 0.129
BMC, whole, kg	2.14 (0.02)	2.18 (0.02)	2.27 (0.02)	0.000	1st vs 3rd : 0.000
					2nd vs 3rd : 0.001
					1st vs 2nd : 0.496
BMD, whole, g/cm^2	1.11 (0.01)	1.12 (0.01)	1.15 (0.01)	0.006	1st vs 3rd : 0.002
					2nd vs 3rd : 0.014
					1st vs 2nd : 0.590
BMD, spine, g/cm^2	0.96 (0.01)	0.95 (0.01)	0.97 (0.01)	0.430	1st vs 3rd : 0.454
					2nd vs 3rd : 0.195
					1st vs 2nd : 0.152
BMD, pelvis, g/cm^2	1.09 (0.01)	1.11 (0.01)	1.15 (0.01)	0.000	1st vs 3rd : 0.000
					2nd vs 3rd : 0.001

BMC, bone mineral content; BMD, bone mineral density.

[a] Data are expressed as means (SE).

[b] Post hoc analysis using the least significant difference t-test (mean difference between two groups).

eTable 2. The correlation between epicardial fat thickness or body mass index difference and bone mineral content difference in MZ and DZ/Sib

	DZ/Sib difference analysis		MZ difference analysis	
	BMC difference	*P*-value	BMC difference	*P*-value
Male	(132 pairs)		(38 pairs)	
Epicardial fat difference	0.244	0.005	0.141	0.397
BMI difference	0.319	0.000	0.342	0.035
Premenopausal	(172 pairs)		(71 pairs)	
Epicardial fat difference	0.218	0.004	0.006	0.960
BMI difference	0.467	0.000	0.311	0.008
Postmenopausal	(40 pairs)		(12 pairs)	
Epicardial fat difference	0.110	0.499	0.075	0.826
BMI difference	0.443	0.004	0.282	0.400

BMC, bone mineral content; BMI, body mass index; DZ, dizygotic twins; MZ, monozygotic twins.

[a] Data presented are Spearman's correlation coefficients.

[b] DZ/Sib difference: pooled same-sex dizygotic twins and age-adjusted same-sex sibling pairs, where the pairwise differences in obesity measures were regressed on the differences in the BMC of the same pairs.

[c] MZ difference: same analysis was conducted for monozygotic twin pairs.

eTable 3. Baseline characteristics of the study population according to echocardiographic image quality

Variables	Good image (n=1,198)	Poor image (n=209)	P-value
Age, years	43.0 (13.7)	38.6 (13.7)	0.000
Men	43.8	47.8	0.022
Women			
Premenopausal	38.4	42.1	
Postmenopausal	17.8	10.0	
Epicardial fat thickness, mm	1.90 (0.75)	1.46 (0.67)	0.000
BMC, whole-body, kg	2.21 (0.46)	2.24 (0.46)	0.161
Height, cm	162.9 (10.7)	164.4 (8.7)	0.052
Weight, kg	63.7 (11.9)	61.9 (12.3)	0.039
BMI, kg/m^2	23.7 (3.2)	22.9 (3.5)	0.011
Waist circumference, cm	81.3 (9.2)	78.4 (9.8)	0.000
Waist-to-hip ratio	0.88 (0.16)	0.85 (0.06)	0.039
Fat mass, kg	17.4 (5.5)	15.5 (5.6)	0.000
Trunk fat mass, kg	9.0 (3.4)	7.7 (3.4)	0.000
Head fat mass, kg	1.1 (0.7)	1.0 (0.2)	0.436
Soft lean mass, kg	44.4 (9.6)	44.5 (10.2)	0.940
Skeletal muscle mass, kg	25.9 (6.2)	26.1 (6.6)	0.853
Hypertension, %	13.4	7.2	0.035
Diabetes mellitus, %	4.9	4.3	0.545
Hyperthyroidism, %	1.4	2.4	0.310
Smokers, %	35.7	36.1	0.901
Drinkers, %	74.3	77.4	0.387
Regular exercise, %	37.7	33.2	0.696

BMC, bone mineral content; BMD, bone mineral density.
[a] Data are expressed as means (SD).
[b] Discrete variables were analyzed by χ^2 test.
